# Management of atrial fibrillation in German military aircrew

**DOI:** 10.1186/s12995-023-00383-5

**Published:** 2023-07-24

**Authors:** Norbert Guettler, Stefan Sammito

**Affiliations:** 1 German Air Force Centre of Aerospace Medicine, Flughafenstrasse 1, Cologne, 51147 Germany; 2 Department of Cardiology, Justus Liebig University, University Hospital Giessen, Medical Clinic I, Giessen, Germany; 3 Department of Occupational Medicine, Medical Faculty, Otto Von Guericke University, Magdeburg, Germany

**Keywords:** Atrial fibrillation, Aircrew, Military, Aviation, Aerospace medicine

## Abstract

**Introduction:**

Arrhythmias are one of the most common causes of loss of flying privileges for both military and civilian pilots in the Western World, and atrial fibrillation (AF) is one of the most common arrhythmias worldwide. Aircrew, and particularly pilots, are subject to a unique and exacting working environment, especially in high-performance military aircraft. This manuscript analyzes AF cases in German military aircrew from both a clinical and occupational perspective to point out specific characteristics in this comparatively young, highly selected, and closely monitored group, and to discuss AF management with the aim of a return to flying duties.

**Methods:**

The digital information systems of the German Air Force Centre of Aerospace Medicine (GAFCAM) were searched for aircrew (pilot and non-pilot aircrew from German Air Force, Army, and Navy) with the diagnosis of AF. Evaluation results for underlying disease, AF characteristics, important clinical findings, and occupational decisions were analyzed in the light of current clinical guidelines and aeromedical regulations.

**Results:**

In a 34-year period, between March 1989 and January 2023, 42 aircrew with at least one episode of AF were registered, all of them were male. The median age at initial diagnosis was 47 years (min 22 years, max 62 years). The median follow-up period was 5.35 years. 19 of them (45%) were pilots. The breakdown of events and occurrence was found to be: single (23), paroxysmal (16), persistent (2), permanent (1). In 27 aircrew (64%) AF terminated spontaneously. Long-term recurrence prevention was variable with catheter ablations in 8 cases. 36/42 aircrew were returned to flight status with restrictions, while 6/42 were permanently disqualified from flying.

**Conclusion:**

Management of AF in military aircrew requires a comprehensive approach regarding the flight environment as well as clinical guidance. Aeromedical disposition should be case-by-case based on aeromedical regulations, individual clinical findings, and specific occupational requirements in this challenging field of work.

## Introduction

Cardiovascular disease is the most common reason for loss of flying privileges for both military and civilian pilots in Western Europe, and cardiac arrhythmias are the main disqualifier [13, 21, 26]. Atrial fibrillation (AF) is the most common arrhythmia worldwide [1, 20, 32]. The global prevalence of atrial fibrillation (AF) has increased substantially over the past three decades and is currently approximately 60 million cases [4, 18]. Currently, >11 million patients are estimated to have AF in Europe, and the total healthcare costs of AF account for ≤2.6% of total healthcare expenditure in Europe [29].

The pathophysiology of AF is characterized by a complex interplay of triggers, perpetuators, and substrate development [14]. Risk factors for AF include increasing age, hypertension, increased pulse pressure, diabetes mellitus, coronary artery disease, myocardial infarction, heart failure, obesity, obstructive sleep apnea, smoking, alcohol use, hyperthyroidism, family history, and certain genetic variants [14, 16]. Stimulant use, excessive alcohol and caffeine intake should also be explored [10].

Aircrew, and particularly pilots, are subject to a unique and exacting working environment, especially in high-performance military flying [23]. In addition to the inherent cognitive demands placed on aircrew, additional factors including hypoxia, acceleration forces in high-performance flight, operational pressure, enemy action, and circadian disruption must be taken into consideration. Most fixed-wing commercial pilots work in a dry, contained environment, pressurised at 6,000 to 8,000 feet. Military high-performance aircraft are far less pressurised to reduce stress on air frame and to provide for an unplanned decompression. Pilots have to breath supplemental oxygen through oxygen masks, sometimes with an increased pressure. Such positive pressure breathing is also used as a countermeasure against high gravitational forces in manoeuvres such as pulling out of a dive or into an inside loop [12, 23]. These exceptional stressors can act as triggers for AF and other arrhythmias. Aeromedical concerns of AF include palpitations, dizziness, syncope, shortness of breath, hemodynamic instability, and stroke (United States Air Force, Medical Service 2023) [28].

This manuscript decribes the incidence, course, management, and occupational consequences of AF in German military aircrew over a 34-year period. AF cases in this highly selected and regularly screened collective are chacterized in comparison with the normal population. Evaluation for underlying disease and treatment of AF are compared to the recommendations by current clinical guidelines, highlighting changes in the management of AF during the observation period. Occupational decisions are discussed in the light of aeromedical regulations by different authorities.

## Methods

The digital information systems (DIL2006, Noris Ingenieurbüro GmbH, Nürnberg, Germany, until October 2020, and sOne, SAmAs GmbH, Paderborn, Germany, from November 2020 on) of the German Air Force Centre of Aerospace Medicine (GAFCAM) were searched for aircrew (pilot and non-pilot aircrew from German Air Force, Army, and Navy) with the diagnosis of AF in a 34-year period, between March 1989 and January 2023. Search criteria were International Classification of Disease (ICD)-10 codes I48.0, I48.1, I48.2, and I48.9. In addition, paper records were searched for relevant information including medical reports by the treating physicians. All German military pilot applicants, licensed non-pilot aircrew applicants, and air traffic controller (ATCO) applicants are medically screened at the GAFCAM. Active-duty German Air Force, Army and Navy pilots and non-pilot aircrew undergo periodic medical examination (PME) at GAFCAM to identify any potential flight disqualifying health conditions as per the German flight requirements. Those found without disqualifying conditions are then deferred for 3 years from GAFCAM, but are evaluated by local flight surgeons annually, up to the age of 40. Pilots above 40 years of age are examined annually at the GAFCAM. Licensed non-pilot aircrew and ATCO are examined annually by the local flight surgeon regardless of their age.

In cases of documented AF, aircrew were examined for cardiac and non-cardiac causes with echocardiography, Holter monitoring, exercise ECG (when in sinus rhythm), and laboratory investigations including electrolytes and thyroid hormones. Those over 40 years of age were examined for coronary artery disease (CAD). This was done by invasive coronary angiography in the early years, within the last decade evaluation of the coronary arteries was mainly performed by computed tomography coronary angiography (CTCA).

Statistical analyses were conducted using IBM SPSS Statistics for Windows 24 (IBM Corp. Released 2016, Armonk, NY, USA). Data analysis was primarily descriptive. Because the Kolmogorov-Smirnov test showed that none of the nominal scale parameters was normally distributed, median with minimum and maximum values were calculated.

According to the regulations of the North Rhine Medical Association, the responsible authority for this study, a vote of the ethics committee was not necessary for this retrospective analysis.

## Results

In a 34-year period, 42 aircrew with at least one episode of AF were registered, all of them were male. The median age at initial diagnosis was 47 years (min 22 years, max 62 years), the median body mass index (BMI) was 25.8 kg/m^2^ (min 20.5 kg/m^2^, max 30.5 kg/m^2^). The median follow-up period was 5.35 years (min 0 years, max 20.27 years). Most of the analyzed individuals had either a single episode of AF or paroxysmal AF. Only two had persistent, and one had permanent AF. The latter wanted to participate in aeromedical evacuation (AE) missions. AF patterns, Aircrew roles, and underlying or concomitant disease are shown in Table 1. AF treatment and aeromedical disposition are described in Table 2.

**Table 1:** AF characteristics, aircrew roles, underlying or concomitant disease, treatment of AF, aeromedical disposition, and duration of follow-up of 42 aircrew with AF within a 34-year-period examined at the GAFCAM

	**n**	**%**
AF pattern		
Single episode	23	55
Precipitant identifiable	12	29
Paroxysmal	16	38
Persistent	2	5
Permanent	1	2
Aircrew roles		
Pilots	19	45
Jet (High-Performance)	5	12
Fixed wing	6	14
Rotary wing	8	19
Weapon System Officers	2	5
Operators	1	2
Other non-pilot aircrew	19	45
ATCO	1	2
Underlying or concomitant disease		
Hypertention	12	29
Coronary artery disease	4	10
Diabetes mellitus	1	2
OSAS	2	5
Myocarditis	2	5
Other infection	6	14
ASD II with occlusion	1	2
Mild mitral valve prolapse without regurgitation	1	2

*AF* Atrial fibrillation, *GAFCAM* German Air Force Centre of Aerospace Medicine, *ATCO* Air traffic controller, *OSAS* Obstructive sleep apnea syndrome, *ASD* Atrial septal defect

**Table 2:** AF treatment and aeromedical disposition of 42 aircrew with AF within a 34-year-period examined at the GAFCAM

	**n**	**%**
Acute AF treatment		
Spontaneous conversion	27	64
Pharmacological cardioversion(s)	5	12
Electrical cardioversion(s)	7	17
Cardioversion recommended before end of follow-up	2	5
Electrical Cardioversions not successful	1	2
Longterm rhythm control		
None	14	33
Antiarrhythmic drugs	4	10
Betablockers	24	57
Catheter ablation	8	19
CHA_2_DS_2_VASc Score at initial diagnosis		
0	28	67
1	12	29
≥ 2	2	5
CHA_2_DS_2_VASc Score at the end of follow-up		
0	22	52
1	17	40
≥ 2	3	7
Anticoagulation		
None	29	69
VKA	5	12
DOAC	8	19
Aeromedical disposition		
Fit	3	7
Permanently unfit	6	14
Temporarily unfit	1	2
Fit with waiver		
Without restrictions	22	52
With restrictions	10	24

*AF* Atrial fibrillation, *GAFCAM* German Air Force Centre of Aerospace Medicine, *CHA*_*2*_*DS*_*2*_*VASc* Congestive heart failure, hypertension, age ≥ 75 (doubled), diabetes, stroke (doubled), vascular disease, age 65 to 74 and sex category (female), *VKA* Vitamin K antagonist, *DOAC* Direct oral anticoagulant

More than half of AF cases in this study were single episodes with identifiable precipitants in more than 50% of these. These precipitants were three cases of mental stress, four cases of infections including one myocarditis, one episode during an exercise ECG, one during a centrifuge run, two episodes after alcohol use, one of them in combination with an opulent meal, and one case of post-interventional AF after an occlusion of an atrial septal defect. One aircrew had a syncope when driving his car and was then diagnosed with AF. None of the aircrew in our cohort was reported to have noticed AF symptoms at work or during flying.

Three of five affected high-performance jet pilots, who were older than 50 years of age at initial diagnosis, flew irregularly to keep up their license. 13 non-pilot aircrew were not licensed aircrew and flew irregularly, eight of them were physicians (flight surgeons and AE personnel) with an average age of 52.5 years at initial diagnosis. The symptomatology of AF was mild in most individuals, while in some cases with short AF episodes the symptomatology remained unclear. Four aircrew were known to be asymptomatic with AF being an incidental finding. In total, 28 aircrew were symptomatic with AF, two additional individuals reported unspecific symptoms including weakness and dyspnea in one case, and subsultus in the other one. Six aircrew with AF were asymptomatic, in another six aircrew symptomatology was not reported.

The most frequent underlying risk factor of AF was hypertension. Of the four CAD cases, two received percutaneous coronary interventions (PCI) with stenting, the others had coronary artery stenoses not leading to ischemia.

Most of the AF episodes in our cohort terminated spontaneously. Twelve were cardioverted electrically or pharmacologically. In one case electrical cardioversion was not successful, so this case was declared as permanent AF. For long-term treatment, beta receptor blockers were administered to more than half of all individuals. Class I or III antiarrhythmic drugs according to Vaughan Williams classification (drugs affecting sodium or potassium channels) were used for long-term recurrence prevention in four individuals, catheter ablation in eight individuals. All of them received pulmonary vein isolation (PVI) as primary procedure, one of them in combination with a left atrial roof line. Two of the ablated individuals underwent subsequent ablations at four and five years after the first ablation, respectively. In one of these cases, another procedure is currently discussed due to a recent recurrence. In three individuals of our collective, AF was associated with typical atrial flutter. They received an ablation of the cavotricuspid isthmus. The earliest catheter ablations for AF or atrial flutter were performed in 2006. In one individual, an associated atrial tachycardia was ablated, in another one an accessory pathway.

The CHA_2_DS_2_VASc (congestive heart failure, hypertension, age ≥ 75 (doubled), diabetes, stroke (doubled), vascular disease, age 65 to 74 and sex category (female)) score [19] in our collective was predominantly 0 or 1 (mostly for hypertension). Only three aircrew had a CHA_2_DS_2_VASc score of 2 or more, two of them had sustained a transient ischemic attack, one had hypertension plus diabetes. 13 aircrew were anticoagulated, five with vitamin K antagonists, three of those only temporarily. Eight were anticoagulated with direct oral anticoagulants (DOAC), four of those temporarily. Temporary anticoagulation after catheter ablation for at least two months is mandatory due to the thrombogenicity of the ablation lesions. The first individual anticoagulated with a DOAC received dabigatran in 2011, the others were administered with DOACs 2015 or later.

According to German military regulations, aircrew with AF are usually unfit for flying [17]. After a disqualifying condition has been identified, an aeromedical waiver would be required to return to flight duties that may or may not require restrictions. Three out of 42 analyzed aircrew remained fit for flying (without a waiver), one was temporarily unfit but returned to flying without a waiver, and six were permanently unfit until they quit their services in the German Armed Forces. Two of them were former jet pilots at age 53 and 54, two were helicopter pilots at age 49 and 56. These four pilots had been flying irregularly just to keep up their license and stopped flying after AF diagnosis and treatment. A non-pilot aircrew at age 53 retired after reaching his retirement age. The last one was a 23-year-old transport pilot diagnosed with AF in June 2022. At the end of the follow-up period, he was temporarily unfit because of a different disease and was awaiting a waiver after convalescence. 32 were fit with a waiver, eleven of them with restrictions, 21 without restrictions. Ten of the eleven restrictions were given to pilots and were mostly “operational multi-pilot limitation (OML)” or “no high-performance”.

## Discussion

We analyzed all cases with AF in the German Armed Forces over a 34-year period from a clinical and occupational perspective. To the best of our knowledge, this is the largest analysis of AF in military aircrew in Europe. The United States Air Force Aerospace Waiver Guide Compendium mentions a five-year review by the Aeromedical Information Management Waiver Tracking System through 2020 including 76 cases of AF/atrial flutter, 10 of which were disqualified. According to the same source, there is an Aeromedical Consultation Service Atrial Fibrillation Working Group currently following 168 cases [28]. In 2013, Hunter et al. published a five-year review of AF in the British Royal Air Force with 23 AF cases [15]. Military aircrew are highly selected for medical fitness and occupational aptitude, and they are regularly screened for disease incompatible with flight safety. The incidence of AF is therefore much lower than in an unselected general population [2, 9, 18]. Regarding record identification numbers and yearly examination numbers, it can roughly be estimated that between 20,000 and 25,000 aircrew were examined at the GAFCAM in the analyzed 34-year period. Out of these, 42 had AF. The incidence of AF increases with age [22], and none of the analyzed aircrew was older than 62 years at initial diagnosis. Besides differences in AF incidence, the peculiarity of the analyzed collective also led to differences in AF characteristics. Most of our cases were single AF episodes, often caused by an identifiable precipitant, or paroxysmal AF. Stroke risk was mostly low, and only few aircrew needed permanent anticoagulation.

Aeromedical concerns of AF include palpitations, dizziness, shortness of breath, presyncope, syncope, exercise intolerance, haemodynamic instability, and stroke risk. The loss of atrial contribution to cardiac output, loss of atrioventricular synchrony, and the rapid ventricular response during an episode may impair cardiac performance, especially during exertion, and can be acutely distracting or incapacitating [10].

Aeromedical regulations for aircrew with AF are complex and vary between civilian and military licensing authorities of different countries. According to European Union regulations [5] professional pilots (class 1) with AF must be referred to the licensing authority, private pilots (class 2) must be assessed in consultation with licensing authority. For initial class 1 and ATCO applicants, a fit assessment should be limited to those with a single episode of arrhythmia which is considered by the medical assessor of the licensing authority to be unlikely to recur. For revalidation, applicants may be assessed as fit, class 1 pilots with an OML, if cardiological evaluation is satisfactory and the stroke risk is sufficiently low [7]. Anticoagulation is allowed with vitamin K antagonists and with direct oral anticoagulants (DOAC). Class 2 applicants with AF may be assessed as fit if cardiological evaluation is satisfactory and the stroke risk is sufficiently low. In case of anticoagulation with a vitamin K antagonist a safety pilot limitation is required. If anticoagulated with DOACs, such a limitation is not necessary. After catheter ablation class 1 pilots are primarily unfit for flying. A fit assessment may be considered following successful catheter ablation and should require an OML for at least one year, unless an electrophysiological study, undertaken at a minimum of two months after the ablation, demonstrates satisfactory results. For ATCO a fit assessment may be considered after a minimum of two months following successful catheter ablation provided an electrophysiological study has demonstrated satisfactory results [6]. For class 2 pilots a fit assessment may be considered following successful catheter ablation subject to satisfactory cardiological review undertaken at a minimum of two months after the ablation [7].

According to the regulations issued by the US Federal Aviation Authority (FAA) in most cases a deferral and a FAA decision is required. The aeromedical examiner (AME) has to provide detailed information by a Non-Valvular Atrial Fibrillation Initial or Recertification Status Report. In case of a positive decision a Special Issuance is issued by the FAA [8].

In the German Armed Forces all aircrew with atrial fibrillation with or without catheter ablation are unfit for flying [17]. Based on an individual decision, a waiver can be granted by the GAFCAM.

In the US Air Force, a history of atrial fibrillation is disqualifying for all flying classes and retention. The one exception is a single episode of atrial fibrillation clearly associated with a reversible cause. Additionally, the use of maintenance medications for the treatment or prevention of major rhythm disturbances including atrial flutter or atrial fibrillation requires a waiver for retention and all flying classes. A history of catheter ablation is also disqualifying for all flying classes [28]. Waivers for recurrent AF without hemodynamic symptoms may be granted for trained aircrew, but not for initial pilot applicants.

All these regulations focus on several criteria which are important for disposition. First of all, underlying or concomitant cardiac or non-cardiac disease that may be incompatible with flying has to be excluded by thorough evaluation. The detection of other arrhythmias associated with AF is also important. As some of the regulations allow for flying in case of a single episode with an identifiable cause, triggers or precipitants of single AF episodes have to be identified and the likelihood of recurrence estimated. As AF is a progressive disease [3, 24], it has often been discussed if freedom of recurrence after single episodes is realistic. But in our collecive, this was frequently observed.

In Europe, symptom severity is often characterized using the European Heart Rhythm Association (EHRA) symptom scale [14, 30]. Symptoms (palpitations, fatigue, dizziness, dyspnea, chest pain, and anxiety during AF) are evaluated with regard to how they affect the patient´s daily activity. EHRA 1 would mean no symptoms, EHRA 2a means mild symptoms (normal daily activity not affected), EHRA 2b moderate symptoms (normal daily activity not affected, but patient troubled by symptoms), EHRA 3 means severe symptoms (normal daily activity affected), and EHRA 4 means disabling symptoms (normal daily activity discontinued). Characterization of symptom severity is not only helpful for treatment decisions, but also for aeromedical assessment, e. g. if recurrence prevention should fail in the long term.

Common stroke risk factors are summarized in the clinical risk-factor-based CHA_2_DS_2_VASc score [19]. In our exclusively male collective, a score of 0 meant that permanent anticoagulation was not required, with a score of 1 it had to be considered, if ≥ 2 anticoagulation was mandatory. But stroke risk scores have to balance simplicity and practicality against precision. For many risk factors, stroke risk is a continuum rather than an artificial low-, moderate- or high-risk category. It has been shown that in the definition of “vascular disease”, angiographically significant CAD [27] and complex aortic plaque in the descending aorta [31] should also be included as a risk factor. This, however, does not occur in many tables. CHA_2_DS_2_VASc score results are dynamic and change over time, regular reevaluations are required.

DOACs have entered EASA regulations in 2019, before only vitamin K antagonists were allowed for anticoagulation. In a meta-analysis DOACs have shown to be non-inferior in the prevention of stroke compared to warfarin, but they were associated with a 10% reduction of all-cause mortality, a non-significant (14%) reduction in major bleeding risk, significant (52%) reduction in intracranial haemorrhage, but 25% increase in gastrointestinal bleeding [25]. In many air forces, anticoagulation is not compatible with military flying because of the increased risk of injuries in combat scenarios and the sometimes impaired medical treatment in missions abroad.

For more than ten years now, catheter ablation has been accepted as a first-line treatment for rhythm control alternative to drug treatment. It is often preferred by aircrew as many side effects of antiarrhythmic drugs are incompatible with flying [10, 11]. After catheter ablation for AF, however, recurrences requiring reablation are not rare and may occur even years after primary procedure. One year of OML or, alternatively, a diagnostic electrophysiological testing after a minimum of two months to ensure ablation success, as recommended by EASA, are therefore not always helpful.

The commercialization of space is moving forward and will increasingly provide opportunities for older and less selected individuals to fly into space. Many of these individuals will be at increased risk of cardiovascular disease or arrhythmia. Knowledge about the pathophysiology and triggers of AF under the conditions of such flights as well as management of the arrhythmia will therefore be of increasing importance. Further studies on AF in environments comprising acceleration forces, possible hypoxia etc., and including older individuals and a higher ratio of females are warranted.

There are some strengths and limitations of our study that have to be mentioned in this manuscript. One of the strengths is the comprehensive analysis of a large number of aircrew over a long duration. In addition to the very long observation time, it is also a strength that there is only one center in the German Armed Forces for aeromedical assessment of military aircrew of all services (Air Force, Army, and Navy). So, the analysis included every aircrew with AF within the last 34 years. On the other hand, it is a limitation that the total number of aircrew examined in the 34-year period is not exactly known. Therefore, the incidence cannot be calculated, but it is certainly much lower than in the general population. Another limitation is that only German data were analyzed. Combining data from other countries’ military flying health data repositories might bolster the sample size. Additionally, this study only provided data on male aircrew, as only males were affected by AF during the observation period. It is not clear if databases of licensing authorities worldwide, e. g. the FAA, have been reviewed to exclude or include females as part of their comparisons and critical thoughts.

## Conclusion

In conclusion, the incidence of AF in the comparatively young, male, highly selected, and medically monitored group of aircrew is, as expected, much lower than in the general population. Management of AF in military aircrew requires a comprehensive approach regarding military aviation environment as well as clinical guidance. Aeromedical disposition should be case-by-case decisions based on aeromedical regulations, individual clinical findings, and specific occupational requirements in this challenging field of work.

## Data Availability

The data that support the findings of this study are available from the Federal Ministry of Defense. Data are available on reasonable request.
